# Inflammation-inducible promoters to overexpress immune inhibitory factors by MSCs

**DOI:** 10.1186/s13287-023-03501-6

**Published:** 2023-09-23

**Authors:** Anton Selich, Jenni Fleischauer, Tina Roepke, Luisa Weisskoeppel, Melanie Galla, Constantin von Kaisenberg, Ulrich A. Maus, Axel Schambach, Michael Rothe

**Affiliations:** 1 Hannover Medical School, Institute of Experimental Hematology, Building J11, HBZ, Level 01, Room, 6540 Hannover, Germany; 2 Division of Experimental Pneumology, Hannover Medical School, Hannover, Germany; 3 Department of Obstetrics and Gynecology, Hannover Medical School, Hannover, Germany; 4 German Center for Lung Research, Partner Site BREATH, Hannover, Germany; 5 Division of Hematology/Oncology, Boston Children’s Hospital, Harvard Medical School, Boston, MA USA

**Keywords:** Mesenchymal stromal cells, Immune inhibition, Genetic engineering, Gene regulation

## Abstract

**Background:**

Mesenchymal stromal cells (MSCs) are excessively investigated in the context of inflammation-driven diseases, but the clinical results are often moderate. MSCs are naturally activated by inflammatory signals, which lead to the secretion of immune inhibitory factors in inflamed tissues. Many work groups try to improve the therapeutic outcome of MSCs by genetic modification and the constitutive overexpression of immune modulatory transgenes. However, the ectopic secretion of immune inhibitory transgenes increases the chances of infections, and constitutive transgene expression is not necessary for chronic diseases undergoing different inflammatory stages.

**Methods:**

We designed and tested inflammation-induced promoters to control transgene expression from integrating lentiviral vectors in human umbilical cord MSCs. Therefore, we investigated different combinations of general transcription factor elements to achieve a minimal promoter with low basal activity. The best candidates were combined with interferon-induced GAS or ISRE DNA motifs. The constructs with the highest transgene expression upon addition of pro-inflammatory cytokines were compared to vectorized promoters from inflammation-induced genes (CD317, CXCL9, CXCL10, CXCL11 and IDO1). Finally, we investigated IL10 as a potential immune inhibitory transgene by transcriptome analyses, ELISA and in an acute lung injury mouse model.

**Results:**

The synthetic promoters achieved a high and specific transgene expression upon IFN-γ addition. However, the CXCL11 promoter showed synergistic activity upon IFN-γ, TNF-α and IL1-β treatment and surpassed the transgene expression height of all tested promoters in the study. We observed in transcriptome analyses that IL10 has no effect on MSCs and in ELISA that IL10 is only secreted by our genetically modified and activated CXCL11-IL10-MSCs. Finally, transplanted CXCL11-IL10-MSCs increased CD19+ and CD4+ lymphoid cells, and decreased CD11b+ Ly6g myeloid cells in an ALI mouse model.

**Conclusion:**

These results provide new insights into MSC inflammatory activation and the subsequent translation into a tool for a tailored expression of transgenes in inflammatory microenvironments. The newly developed promoter elements are potentially interesting for other inflamed tissues, and can be combined with other elements or used in other cell types.

**Supplementary Information:**

The online version contains supplementary material available at 10.1186/s13287-023-03501-6.

## Background

Mesenchymal stromal cells (MSCs) are excessively investigated as a therapeutic option for a broad spectrum of inflammatory diseases in animals and patients [1]. However, many trials show only a limited beneficial effect upon MSC application leading to only a few regulatory authority-approved therapies worldwide [2–4]. To enhance the natural MSC effects, many work groups use genetic modifications to overexpress factors contributing to immune modulation (TGF-β, IL10), migration to inflammatory microenvironments (CXCR4), and other mechanisms [5–8]. However, MSCs are initially entrapped in the lung, and the subsequent migration is a complex and not fully understood process [9, 10]. It was shown that the constitutive presence of immune inhibitory factors in ectopic organs could increase the risk of bacterial infections or autoimmune-like observations [11, 12]. Additionally, some diseases, such as rheumatoid arthritis, can occur in flares [13], making responsive immune inhibition preferable. MSCs increase their immune inhibitory ability upon contact with pro-inflammatory cytokines like IFN-γ, TNF-α, and IL1-β [14], and a constitutive transgene expression could diminish the advantage of a responsive therapy. Therefore, we tested whether pro-inflammatory cytokines could trigger transgene expression in genetically modified MSCs. Several natural promoters are activated by specific extracellular signals or the activation of biological pathways. Synthetic promoters are composed of pathway-specific transcription factor binding sites and a minimal promoter, which contains general transcription factor binding sites to help assemble the RNA polymerase complex [15]. The design of natural and synthetic promoters can be very challenging. Some minimal promoters induce transgene expression without the activation signal (leakiness) or achieve only low transgene expression [16]. Physiological gene regulation often depends on regulatory elements, which can be distributed over a range of more than 20 kb and can be challenging to vectorize [17]. As not all cells are equally susceptible to an activation signal, promoter designs have to be evaluated in the respective target cell.

To stably introduce our promoter candidates and the respective transgene cassettes into human umbilical cord MSCs (UC-MSCs), we used 3rd generation lentiviral vectors harboring an eGFP under the control of our promoter candidates. In the same constructs, a constitutively active human phosphoglycerate kinase (PGK) promoter drove the expression of mCherry, serving as a transduction control. We designed vectors with seven different combinations of typical minimal promoter elements, such as a GC-box, CAAT-box, TFIIB recognition element (BRE), TATA-box, motif ten element (MTE) and downstream promoter element (DPE) [15]. In a second step, we combined the seven minimal promoter candidates with several copies of the gamma interferon activation site (GAS) or interferon-sensitive response element (ISRE), as MSCs in our hands are mainly activated by IFN-γ [18, 19]. The synthetic promoters were compared to natural promoters of genes activated by pro-inflammatory cytokines (CXCL9, CXCL10, CXCL11, CD317, IDO). Finally, we replaced the reporter gene eGFP with IL10 as an exemplary anti-inflammatory cytokine and transplanted the IL10-MSCs into an LPS-induced acute lung injury (ALI) and acute respiratory distress syndrome (ARDS) mouse model. In our proof-of-concept mouse experiments, we showed a modulation of the immune cell composition in the lung. Overall, inflammation-inducible promoters might be especially useful in diseases where constitutive transgene expression is harmful to the cells or the patient.

## Material and methods

### UC and MSC culture

Human UC was obtained from full term deliveries (38–40 weeks) of healthy mothers after written informed consent, in accordance with the standards of the Hannover Medical School Ethics Committee and with the Helsinki Declaration of 1975, as revised in 1983. UC pieces (UCP) were prepared as previously described [19] and cultivated in MSC15 (MEM α, GlutaMAX™ Supplement, no nucleosides (Thermo Fisher Scientific, Waltham, MA, USA), 15% hAB-serum (C.C.Pro GmbH, Oberdorla, Germany), 1% penicillin/streptomycin (PAN-Biotech, Aidenach, Germany)) or human serum replaced by 5% platelet lysate (PL BioScience GmbH, Aachen, Germany). After approximately two weeks, the 12-wells (Sarstedt, Nuembrecht, Germany) contain around 0.5–1.2*10^5^ adherent cells. To expand cells from 12 to 6-well plates (Sarstedt) for subsequent analysis, we removed the UCP and passaged the MSC culture with trypsin (PAN-Biotech). The MSC monolayer cultures were maintained in MSC10 (MEM α, GlutaMAX™ Supplement, no nucleosides (Thermo Fisher Scientific), 10% hAB-serum (C.C.Pro GmbH), 1% penicillin/streptomycin (PAN-Biotech)) or with 5% platelet lysate (PL BioScience GmbH). Mesenchymal origin was confirmed by antibody staining and was characterized as CD34- (Biolegend, San Diego, USA), CD45- (Miltenyi, Bergisch Gladbach, Germany), CD73 + (Biolegend), CD90 + (Biolegend) and CD105 + (Miltenyi).

### Plasmid cloning, vector production, and transduction

To obtain lentiviral vectors with inducible eGFP and constitutive mCherry expression, we used the vector design based on our previously published All-in-one constitutive Anti-GD2 CAR and inducible cytokine vector [20]. We replaced the CBX3.EFS in the RRL.PPT.CBX3.EFS.mCherry.wpre [21] vector by inducible promoter candidates, followed by eGFP.PGK from the pRRL.PPT.NFATenh.synTATA(Merlet).EGFP.PGK.newMCS.EBFP2.i2.ZeoPRE plasmid [20]. The 8xGAS element was kindly provided by Michael Morgan and the 7xISRE element was produced by an overlap PCR (Forward: GCGAAACCGAAACTATAGCGAAACCGAAACTTATGCGAAACCGAAACTGTA, Reverse: TACAGTTTCGGTTTCGCATAAGTTTCGGTTTCGCTACAGTTTCGGTTTCGCAA). The core promoters were cloned by HiFi DNA Assembly (New England BioLabs, Ipswich, MA, USA) of oligonucleotides containing either the desired element or a random sequence. We used the ENSEMBL database for the annotation of physiological promoters [22] and PCR amplified them from human genomic DNA. All promoter sequences are shown in Fasta format in Additional file 1.

Third-generation SIN lentiviral vectors were pseudotyped with VSV-G using a four-plasmid split packaging system and the calcium phosphate precipitation method as previously described [23]. 5 × 10^6^ HEK293T cells were seeded in 10 cm-dish (Sarstedt) in HEK293T medium(DMEM (Thermo Fisher Scientific), 10% FCS (PAN Biotech), 100 U/mL penicillin/100 μg/mL streptomycin (PAA, Coelbe, Germany) and 1 mM sodium pyruvate (PAA)). On the next day, the medium was replaced by HEK293T medium with 20 mM HEPES (PAN Biotech) and 25 µM chloroquine (Sigma-Aldrich, St. Loius, MO, USA). For each plate, 5 µg viral vector, 12 µg pcDNA3.GP.4xCTE [24], 5 µg pRSV-REV (kindly provided by T. Hope) and 1.5 µg pMD.G [25] were precipitated and added to the HEK293T cells. After 8 h, medium was replaced by fresh HEK293T medium. The medium was harvested several times, viral particles pelleted with 82,000xg for 2 h, resuspended in PBS and stored at -80 °C. Since the transduction efficiency of primary UC-MSCs varies, we prepared several vector dilutions in MSC10 with heat-inactivated human serum (56 °C, 20 min) and 4 µg/mL ml protamine sulfate (Sigma-Aldrich). We measured mCherry transgene expression after at least six days with the CytoFLEX S (Beckman Coulter) to assess transduction efficiency and to rule out the influence of transduction efficiency on eGFP expression height after activation. To test the activity of inducible promoter candidates, we added 10 ng/mL IFN-γ (PeproTech, Cranbury, NJ, USA), TNF-α (PeproTech), and IL1-β (PeproTech) alone or in combination for 24 h and measured the eGFP expression by flow cytometry. Flow cytometry data were analyzed with FlowJo 10 (Becton Dickson, Franklin Lakes, NJ, USA) and visualized with the ggplot2 R-package [26]. Statistical significance was calculated with a Welch-Test, and a subsequent Bonferroni corrected pairwise T-test (R Software). The transcription factor binding sites were assessed with AliBaba2.1 [27] and visualized with ggplot2. The expression of CD317 and IDO was checked by surface antibody staining of CD317 (BioLegend) and intracellular staining of IDO (intracellular staining kit from BioLegend and antibody from ThermoFisher).

### Transcriptome analyses

UC-MSC were seeded in MSC10 medium and kept in culture for 24 h to enable attachment. Afterward, cultures were treated for 24 h with different concentrations of recombinant IL10 (PeproTech), detached, harvested by centrifugation at 400xg for 5 min, resuspended in 700 μL RNAzol B reagent (WAK-Chemie Medical, Steinbach, Germany), and frozen at − 80 °C. Total RNA was isolated with the Direct-Zol RNA MiniPrep Kit (Zymo Research) and on-column DNAse treatment. The total RNA was submitted to the Research Core Unit Genomics of Hannover Medical School. The Microarray utilized in this study represents a refined version of the Whole Human Genome Oligo Microarray 4 × 44 K v2 (Design ID 026652, Agilent Technologies), called ‘026652QM_RCUG_HomoSapiens’ (Design ID 084555) developed by the Research Core Unit Genomics (RCUG) of Hannover Medical School. Microarray design was created at Agilent’s eArray portal using a 1 × 1 M design format for mRNA expression as a template. All non-control probes of design ID 026652 have been printed five times within a region comprising a total of 181,560 Features (170 columns × 1068 rows). Four of such regions were placed within one 1 M region, giving rise to four microarray fields per slide to be hybridized individually (Customer Specified Feature Layout). Control probes required for proper Feature Extraction software operation were determined and placed automatically by eArray using recommended default settings.

200 ng of total RNA were used to prepare aminoallyl-UTP-modified (aaUTP) cRNA (Amino Allyl MessageAmp™ II Kit; #AM1753; Thermo Fisher Scientific, Waltham, MA, USA) applying one round of amplification as directed by the company, except for a twofold downscaling of all reaction volumes. Just one half of the generated cDNA was used for aaUTP-cRNA synthesis. The labeling of aaUTP-cRNA was performed by use of Alexa Fluor 555 Reactive Dye (#A32756; Thermo Fisher Scientific) as recommended in the manual of the Amino Allyl MessageAmp™ II Kit (twofold downscaled reaction volumes).

cRNA fragmentation, hybridization and washing steps were carried-out as recommended in the ‘One-Color Microarray-Based Gene Expression Analysis Protocol V5.7,’ except that 700 ng of each fluorescently labeled cRNA population were used for hybridization.

Slides were scanned using the Agilent Micro Array Scanner G2565CA (pixel resolution 3 µm, bit depth 20). Data extraction was performed with the ‘Feature Extraction Software V10.7.3.1’ by use of the extraction protocol file ‘GE1_107_Sep09.xml’.

The data were analyzed with the limma package and visualized with ggplot2 [28, 29]. As donor variability was strong, we used the donor as a factor in the design matrix, which ensures the comparison within a donor. The data can be accessed in the Gene Expression Omnibus database (GSE224735).

### Functional analyses of the CXCL11S1-IL10 vector

MSC monolayer cultures from three donors were transduced with the lentiviral vectors RRL.PPT.CXCL11S1.eGFP.PGK.mCherry.wpre or RRL.PPT.CXCL11S1.IL10.PGK.mCherry.wpre pseudotyped with VSV-G as described before. All experiments were performed at least six days after transduction to provide time for the dilution of episomal 1- and 2-LTR circles. MSCs were activated with 25 ng/mL IFN-γ, TNF-α, and IL1-β for 24 has described before and analyzed. The IL10 content in the medium supernatant was quantified by ELISA (Thermo Fisher Scientific), and eGFP expression was measured by flow cytometry.

### Acute lung injury mouse model

Animal experiments were approved by the supervising animal research review board at Hannover Medical School, the Lower Saxony State Office for Consumer Protection and Food Safety (21/3773) and followed the ARRIVE guidelines. The experiments and the analyses were performed unblinded. Mice (C57BL/6 J, 10–14 weeks old) were anesthetized by intraperitoneal injection of 50 µl of 3 mg/kg xylazine hydrochloride (Bayer GmbH) and 75 mg/kg ketamine hydrochloride (Albrecht GmbH) dissolved in sterile 0.9% saline. Subsequently, mice received 5 µg or 10 µg LPS in a total volume of 50 µl/mouse intratracheally to induce acute lung injury [30]. After 4 h, PBS or 5*10^5^ MSCs were injected into the tail vein before mice were killed with an overdoses of isoflurane and subsequent removal of blood from the vena cava after an additional 24 h. Bronchoalveolar lavage fluid (BALF) was collected as previously described [31]. The BALF was centrifuged at 300xg for 10 min, the cells were used for antibody staining, and the supernatant was frozen at − 80 °C for Tnf-α ELISA (R&D Systems, Minneapolis, MN, USA). Lungs were cut into small pieces and digested in 1.5 mL of 1 mg/mL collagenase (Gibco/Thermo Fisher Scientific), 100 µg/mL DNAseI (Sigma-Aldrich) and 5 mM MgCl_2_ (Sigma-Aldrich) at 37 °C for 4 h. The digested lung pieces were pushed through a 100 µm mesh (Sarstedt). Cells were harvested, and the immune cell populations were quantified by antibody staining. DAPI staining (Sigma-Aldrich) was used to discriminate between living and dead cells. Different cell populations were characterized by the markers Cd45 (eBioscience/Thermo Fisher Scientific), Cd11c (BD Bioscience), Cd11b (eBioscience), Ly6g (Biolegend), Cd3 (eBioscience), Cd8 (BD Bioscience), Cd4 (Biolegend) and Cd19 (BD Bioscience). No animals or data points were excluded. The data were analyzed by FlowJo 10 and PRISM 6 (GraphPad/ Dotmatics, Boston, MA, USA). Statistical significance was analyzed by a two-way ANOVA with subsequent Bonferroni multiple comparison correction.

## Results

**Fig. 1:**
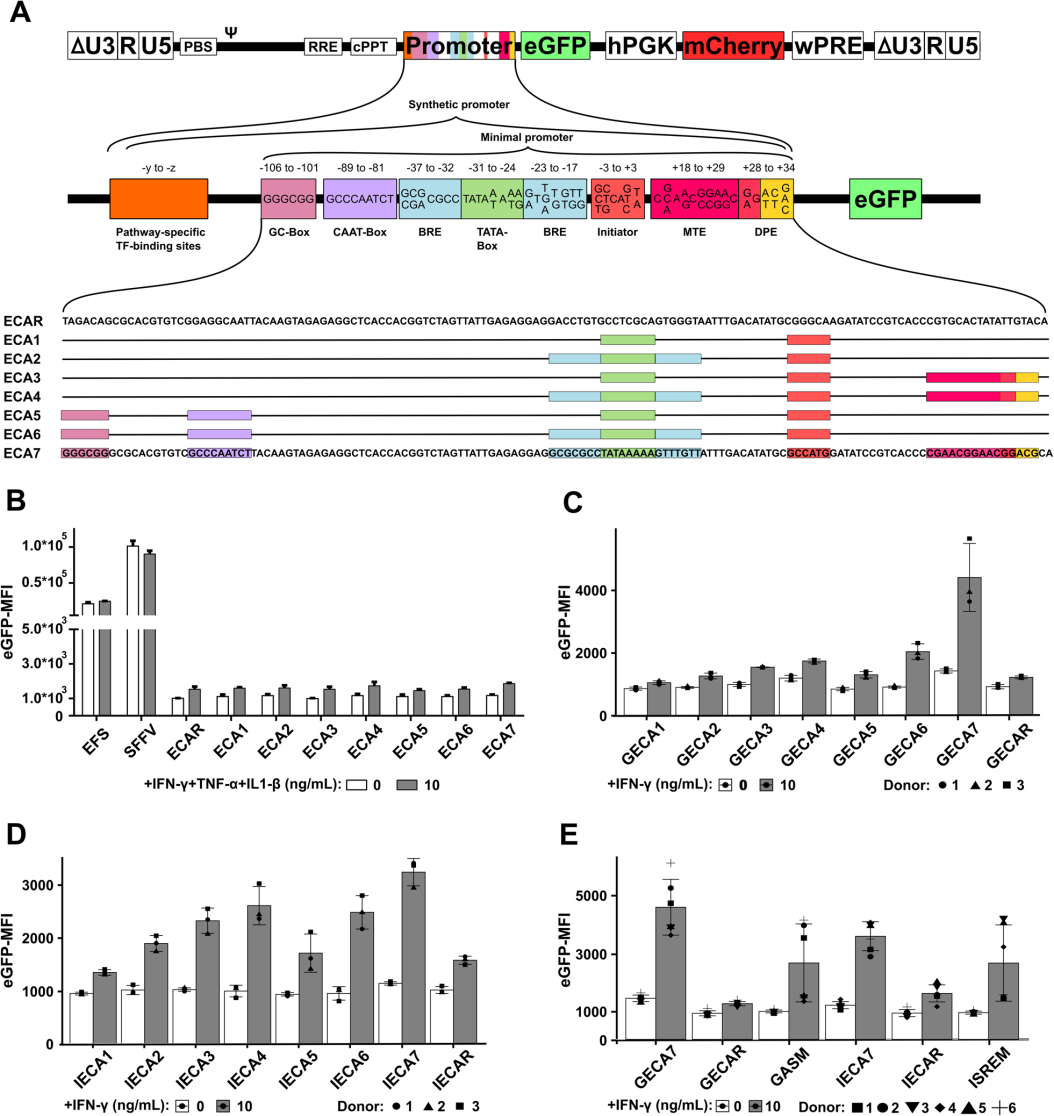
Design and validation of lentiviral vectors and synthetic promoter variants. **A** Design of the third-generation lentiviral vector and minimal promoter candidates. The lentiviral vectors encoded for mCherry under the control of the constitutively active human PGK promoter as transduction control (11–29% mean) and eGFP under the control of one of the synthetic inducible promoter candidates. The synthetic promoters are composed of pathway-specific transcription factor binding sites (orange box) and a minimal promoter. The minimal promoter contains binding sites for different general transcription factors, which are important for the assembly of RNA polymerase complexes but not all present in all physiological promoters. **B** Evaluation of background activity of minimal promoters without pathway-specific transcription binding sites. Transduced human UC-MSCs were activated with IFN-γ, TNF-α and IL1-β (10 ng/mL each). **C** Minimal promoters were combined with gamma interferon activated site (GAS) (box pathway-specific TF-binding sites) and activated with 10 ng/mL IFN-γ. **D** Minimal promoters were combined with interferon-sensitive response element (ISRE) (box pathway-specific TF-binding sites) and activated with 10 ng/mL IFN-γ. **E** Comparison of the best candidates with a previously published synthetic TATA-box [34]. Depicted are the means, and error bars indicate SD (*n* = 3–6 Donors). *U3* unique 3 region, *R* Redundant region, *U5* Unique 5 region, *PBS* Primer binding site, *RRE* Rev responsive element, *cPPT* Central polypurine tract, *eGFP* Enhanced green fluorescent protein, *hPGK* Human phosphoglycerate kinase promoter, *wPRE* Woodchuck hepatitis virus post-transcriptional regulatory element, *BRE* TFIIB Recognition Element, *MTE* Motif Ten Element, *DPE* Downstream Promoter Element, y & z: placeholder for positions depending on the pathways specific transcription factor binding site length, *MFI* Median fluorescent intensity, *ECA* Enhanced core promoter A, *GECA* GAS + Enhanced core promoter A, *GASM* GAS elements combined with the minimal promoter from Merlet et al. [34], *IECA* ISRE + Enhanced core promoter A, *ISREM* ISRE elements combined with the minimal promoter from Merlet et al. [34]. The ANOVA with subsequent Bonferroni-corrected pair-wise *t*-Test were used to calculate statistical significance and are summarized in Additional file 4

We aimed to design inflammation-inducible promoters for UC-MSCs. Conditional regulation of genes by physiological promoters can be very challenging due to a complex architecture [17]. Hence, we designed synthetic promoters, which are usually composed of several repeats of a specific transcription factor binding site and a minimal promoter [16]. Third-generation lentiviral vectors were used to introduce the promoter candidate-driven reporter eGFP (Fig. 1A). High transduction efficiencies might lead to multiple vector copies per cell and higher transgene expression [32]. To allow a fair comparison of promoter strength, all vectors in this study contained the fluorescent protein mCherry, driven by the constitutive hPGK promoter. We applied several vector dosages to MSCs and only compared equally transduced samples (gating strategy in Additional file 2). We analyzed samples below 45% transduction efficiency, as the number of multiple integrations is low [33]. We chose a flow cytomeric read-out instead of PCR, as between the DNA sequence, mRNA and the final protein are many regulatory steps, which we wanted to include.

All minimal promoter candidates contain a TATA-box and a transcription initiator motif. We added various spatially close core promoter elements in blocks around it. A GC-box and CAAT-box as one block (− 106 to − 81 from transcription start site), MTE and DPE as one block (+18 to + 34), and two BREs flanking the TATA-box as one block (− 37 to − 17) (Fig. 1A). These elements replaced, in different combinations, a random nucleotide sequence. The vector design without any promoter elements (enhanced core promoter A random = ECAR) served as a negative control [15]. The various combinations of promoter elements were named enhanced core promoter A (ECA) 1 to 7.

To identify synthetic promoters with low or absent basal activity in UC-MSCs, we introduced the eight combinations (ECA1-7 and ECAR) into CD34-CD45-CD73 + CD90 + CD105 + UC-MSCs with lentiviral vectors (Additional file 3). Transduced cells were treated with 10 ng/mL IFN-γ, TNF-α, and IL1-β. These cytokines were chosen, as they are the most frequently used factors for MSCs activation [14] and the chosen concentration was sufficient to achieve differential transgene expression. Transgene expression from synthetic promoters was compared to expression from the elongation factor 1 α short promoter (EFS) and the spleen focus forming virus (SFFV) U3 promoter, a strong viral promoter [35]. The minimal promoter candidates showed a similar eGFP-MFI as the random nucleotide sequence (ECAR). We only observed autofluorescence of MSCs, which slightly increased upon cytokine treatment (Fig. 1B). The ECA promoters showed no leakiness in the absence of activation signal-specific elements. As MSCs in our hands are mainly activated by IFN-γ [19], all minimal promoter variants were combined with eight copies of the frequently used IFN-responsive element GAS (γ-interferon-activated site) or seven copies of the ISRE (Interferon-sensitive response element) motif (Fig. 1C/D) [18]. The ECA7 variant containing all minimal promoter elements showed the highest eGFP expression in combination with GAS (GECA7) or ISRE (IECA7) upon IFN-γ treatment. Interestingly, the performance of the minimal promoter was different when combined with GAS or ISRE. We compared the GECAR, GECA7, IECAR, and IECA7 to a previously published minimal promoter composed of only the TATA-box “TATATAAT” [34], which was modularly modified by our laboratory [20]. The combinations with the ECA7 promoter showed a similar expression level among six tested UC-MSC donors, whereas the combinations with the Merlet minimal promoter were very variable (Fig. 1E, Additional file 5).

**Fig. 2:**
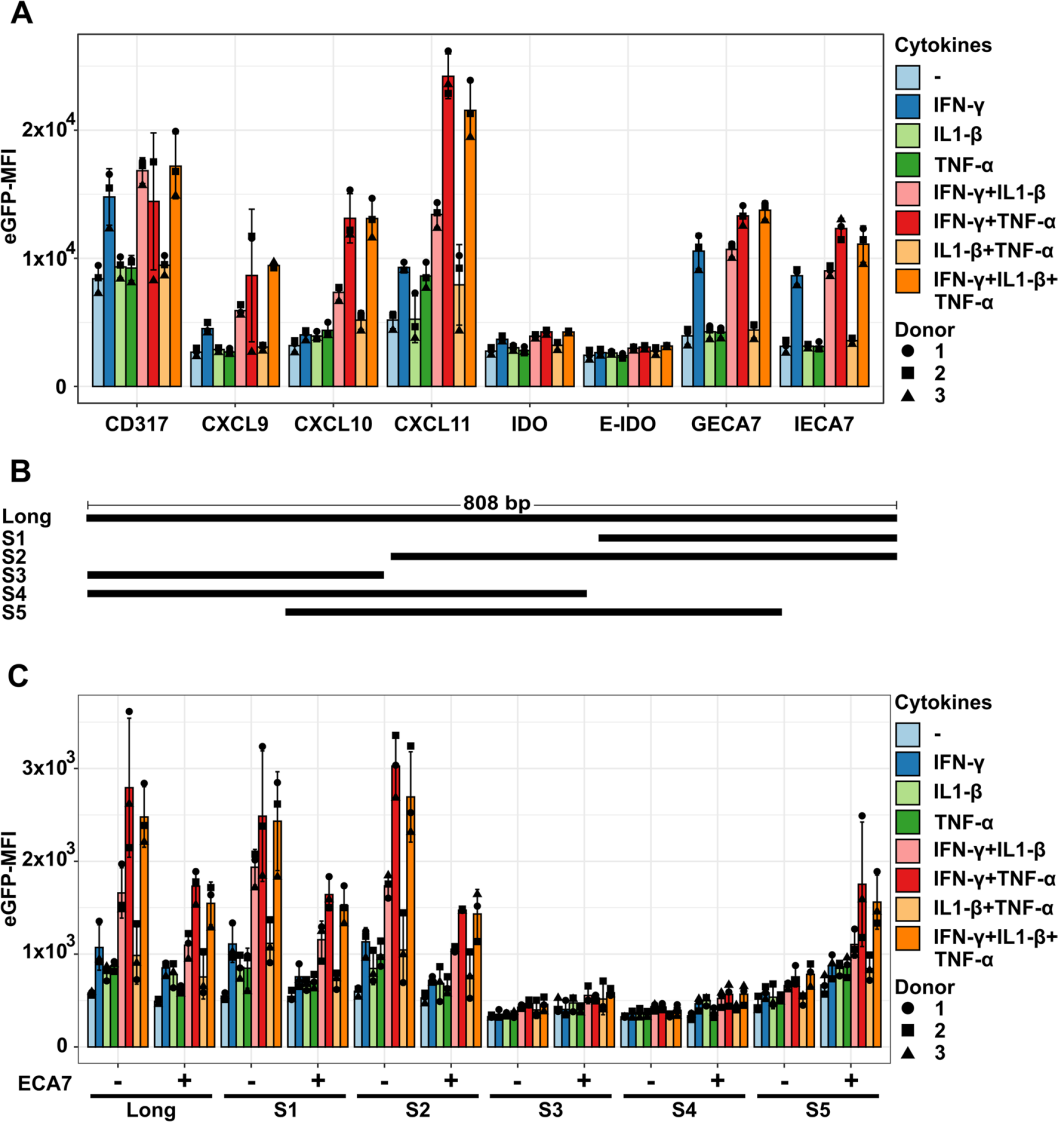
Comparison of synthetic and physiological promoters upon activation with pro-inflammatory cytokines. Human UC-MSCs were transduced with lentiviral vectors coding for eGFP under the control of potentially inflammation-inducible promoters, cultivated in MSC medium (−) or activated with different combinations of pro-inflammatory cytokines (25 ng/mL each). **A** Depicted on the x-axis are the gene names of the physiological promoters CD317, CXCL9, CXCL10, CXCL11, IDO and a spatially close enhancer element in the IDO locus in combination with the IDO promoter (E-IDO). Additionally, the synthetic promoters 8xGAS (GECA7) or 7xISRE (IECA7) in combination with the minimal promoter ECA7 is shown. **B** To identify the most relevant region for the function of the CXCL11 promoter, different shorter versions covering different parts of the CXCL11 promoter were cloned and tested. **C** The newly designed lentiviral vectors were tested in UC-MSCs. Mean transduction efficiency 17–40%. Depicted are the means and SD. The ANOVA with subsequent Bonferroni-corrected pair-wise t Test was used to calculate statistical significance and are summarized in Additional file 4. *bp* Base pair, *ECA7* Enhanced core promoter A7, *GECA7* 8xgamma interferon activated site + enhanced core promoter A7, *IECA7* 7xinterferon-sensitive response element + enhanced core promoter A7, *eGFP* Enhanced green fluorescent protein, *MFI* Median fluorescent intensity

Since an inflammatory microenvironment consists of many regulatory cues and many factors work synergistically, we tested promoters from inflammation-induced genes. Therefore, we used our previously published UC-MSC transcriptomic data to identify genes strongly upregulated by inflammatory cytokines [19]. The promoters of the most upregulated genes *CXCL9*, *CXCL10*, and *CXCL11* were chosen. We used the ENSEMBL database and the predicted promoter regions to replace our synthetic promoter candidates. Furthermore, we chose the promoter of *IDO1* as one of the most prominent MSC effector molecules. We either used it alone or in combination with a closely located enhancer element (E-IDO). We also tested the promoter of BST2/CD317, a reliable MSC activation marker in our hands. The CD317 promoter candidate has a size of 396 bp. Due to a 16 bp GC region, amplification or the commercial synthesis of longer versions was unsuccessful. All vectors with the promoter candidates were transduced into UC-MSCs, activated with different combinations of IFN-γ, TNF-α, and IL1-β, and compared to the synthetic promoters GECA7 and IECA7 (Fig. 2A). An overview of all statistical tests to compare the different promoters is provided in Additional file 5. As expected, the GECA7 and IECA7 promoters were only activated upon IFN-γ treatment. Similar results were observed with the CD317 promoter, but the background expression in the basal MSC medium was already high. The IDO and E-IDO promoter candidates showed a very low transgene expression even upon activation with all cytokines. We checked the activation of CD317 and IDO by surface antibody staining of CD317 and intracellular staining of IDO1 (Additional file 6). We detected CD317 and IDO1 expression upon IFN-γ, IFN-γ + IL1-β, IFN-γ + TNF-α or IFN-γ + IL1-β + TNF-α treatment, which implies that the genes are induced, but our vectorized promoters are lacking important regulatory elements. The CXCL9 promoter was mostly responsive to IFN-γ addition, but TNF-α and IL1-β increased the activity. To achieve expression from the CXCL10 promoter over background, IFN-γ and at least one of the other cytokines were necessary. The CXCL11 promoter was responsive to IFN-γ and TNF-α alone, and the combination led to the highest eGFP expression observed in the experiments. The addition of IL1-β to IFN-γ but not to TNF-α increased the eGFP expression. We chose the CXCL11 promoter for further investigation, as the promoter showed the highest transgene expression and synergistic effects among cytokines.

To better characterize and potentially shorten the CXCL11 promoter, we designed five shorter versions covering different parts of the sequence (Fig. 2B). A short version is preferred as larger vectors usually achieve lower titers, which can hamper clinical translation [36]. Additionally, we tested whether adding the synthetic ECA7 minimal promoter to an inflammation-induced promoter could further boost the activity. The CXCL11 shorter versions S1 (510–809 bp) and S2 (300–809 bp) recapitulate the response of the full-length version (Fig. 2C). The ECA7 addition to these CXCL11 promoter variants decreased the expression height of eGFP. The promoter variants S3 (1–295 bp) and S4 (1–498 bp) showed a markedly decreased activity compared to the full-length promoter, and the activity was not increased/elevated upon ECA7 addition. Interestingly, the S5 version (195–695) showed a substantial reduction in cytokine-induced transgene expression but was partially increased by the addition of the ECA7 promoter downstream. To explain our observations, we looked at transcription factor binding sites (Additional file 7). The S1 and S2 versions contain two close TATA-binding protein (TBP) sites (680–689 & 695–704 bp), whereas S5 lacks the second. The ECA7 addition seemed to restore the core promoter activity in this region. The CXCL11S1 version, which recapitulates the activity of the full-length CXCL11 promoter, contains specific inflammation-regulated transcription factor binding sites, such as NFκB and AP-1 family proteins [37, 38], whereas the inactive S3 and S4 do not contain any. We continued with the CXCL11S1 promoter, as it was the shortest of the potent promoters.

**Fig. 3:**
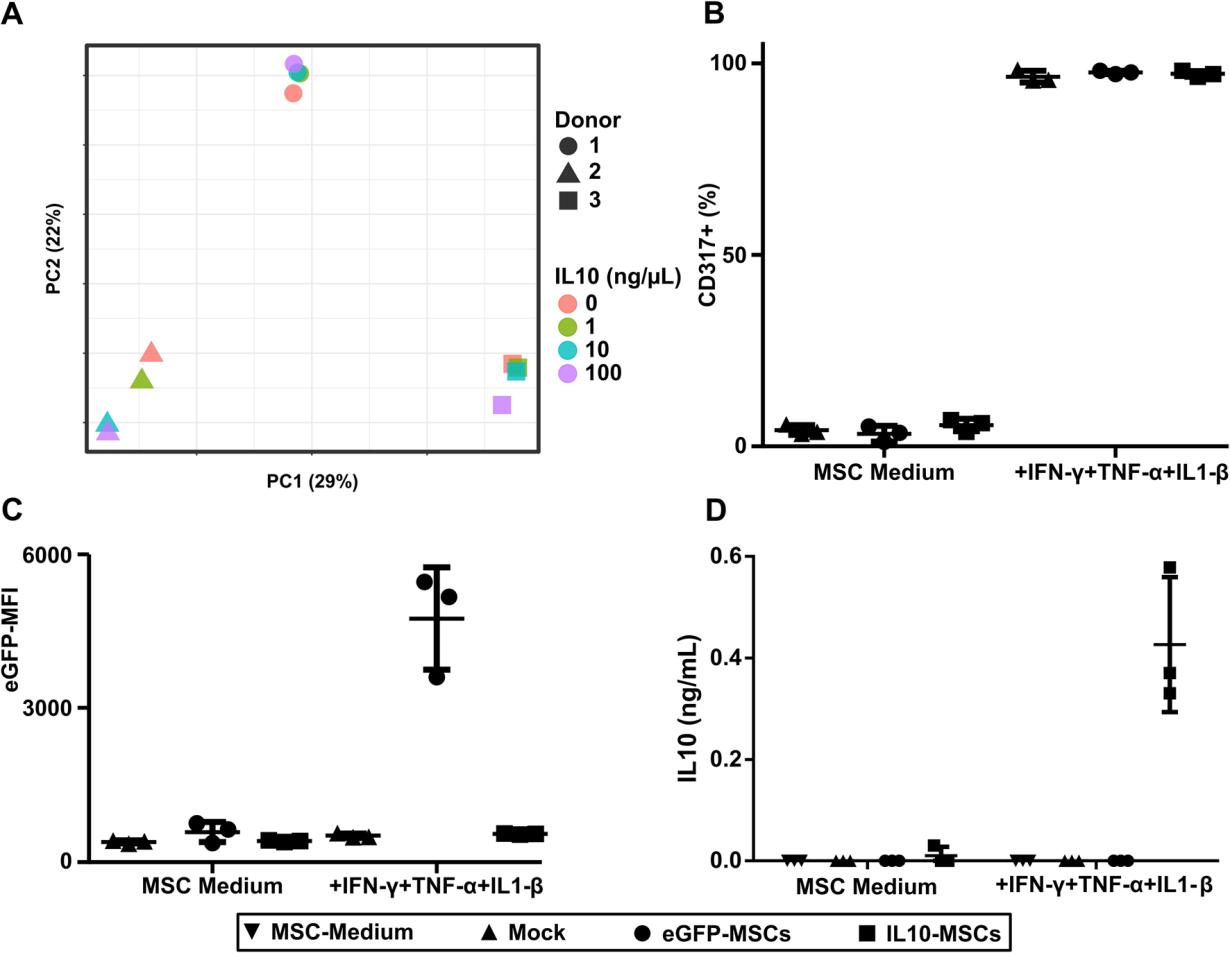
Evaluation of human IL10 as an MSC secreted transgene. **A** UC-MSCs were treated with different concentrations of recombinant human IL10 for 24 h and the gene expression differences were assessed by microarray analysis. No significant differences were measured after IL10 treatment within the same donor. **B** UC-MSCs were either not transduced (Mock), transduced with lentiviral vectors coding for eGFP or human IL10 under the control of the CXCL11S1 promoter. The MSC activation upon treatment with IFN-γ, TNF-α and IL1-β (25 ng/mL each) was controlled by an antibody staining of CD317. **C** The eGFP expression was controlled by flow cytometry. **D** The IL10 amount in conditioned medium was quantified with ELISA. Depicted are the means. Error bars indicate SD. MFI: median fluorescent intensity. The ANOVA with subsequent Bonferroni-corrected pair-wise *t* Test was used to calculate statistical significance and is summarized in Additional file 4

To test whether the CXCL11S1 promoter-driven expression can efficiently induce a therapeutic transgene in an inflammatory setting, we chose the anti-inflammatory cytokine IL10, as it is often used in the context of MSC immune modulation in many diseases [6, 7, 39–43]. Important for our study, the human IL10 can inhibit mouse immune cells [44]. To exclude a negative influence of IL10 expression on MSC immune modulation, we treated MSCs with different concentrations of recombinant IL10 and analyzed the transcriptome (Fig. 3A). No significantly dysregulated genes were identified upon treatment with increasing amounts of IL10. We replaced the eGFP marker gene in our vector with a codon-optimized human IL10 cDNA and transduced human UC-MSCs. The transduction with the eGFP control or IL10 vector did not affect MSC activation, as shown by the expression of the activation marker CD317 (Fig. 3B). Only the eGFP control vector showed increased eGFP expression upon activation with IFN-γ, TNF-α, and IL1-β (Fig. 3C). Vice versa, we detected only the secretion of human IL10 in the supernatant of activated CXCL11S1-IL10-transduced MSCs (Fig. 3D). Importantly, as we detected no IL10 secretion in the supernatants of eGFP control vector-transduced or untransduced MSCs, our IL10 vector expanded the natural secretome of MSCs by an additional immune-modulatory factor.

**Fig. 4:**
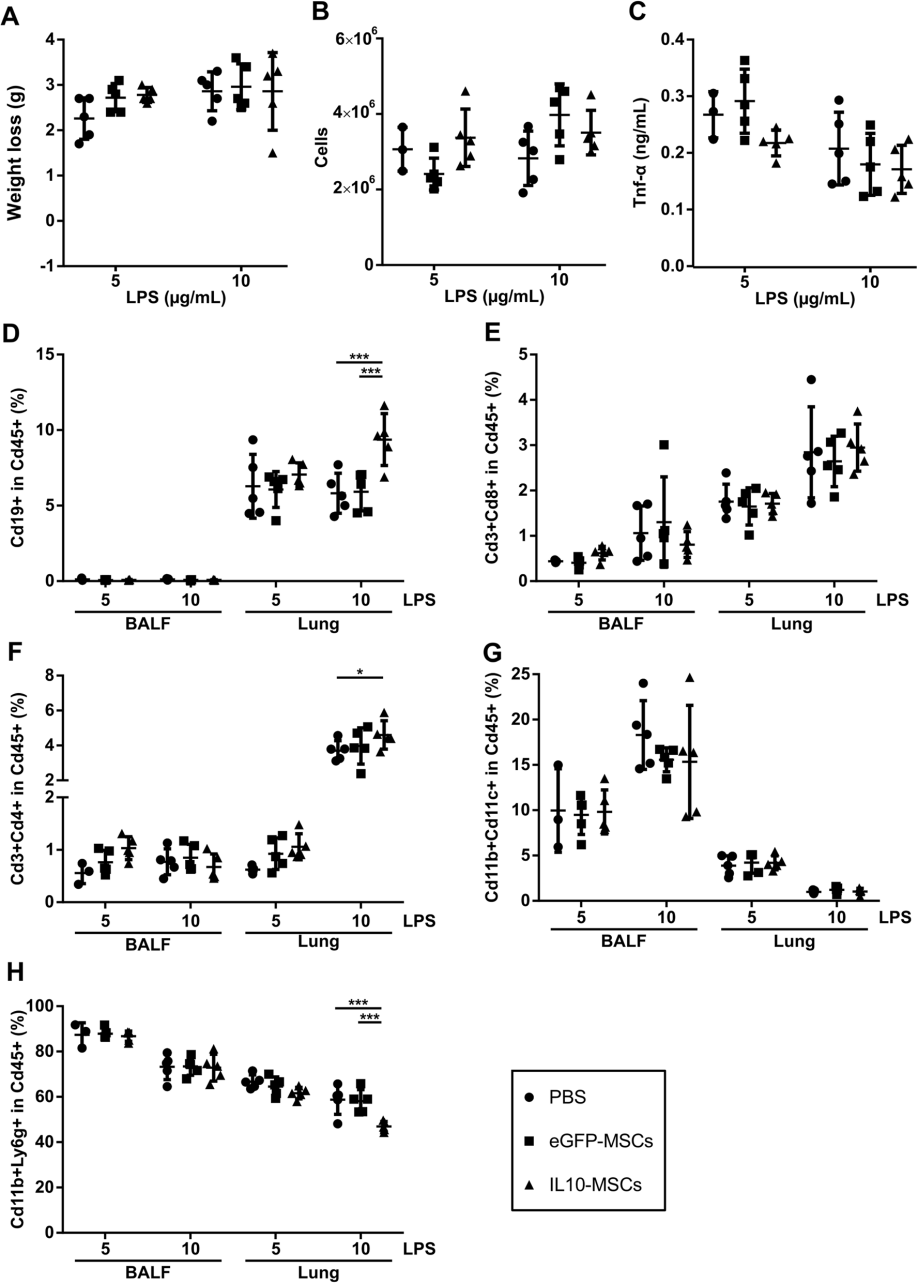
The influence of CXCL11S1-driven IL10 overexpression by UC-MSCs in an ALI mouse model. Human UC-MSCs were transduced with a lentiviral vector coding for a constitutively expressed mCherry driven by a PGK promoter as transduction control. Additionally, the vector coded for eGFP or human IL10 under the control of CXCL11S1 promoter. C57BL/6 J mice were intratracheally treated with 50 µL of 5 µg or 10 µg LPS to induce ALI. After 4 h, PBS or 5 × 10^5^ MSCs either transduced with CXCL11S1-driven eGFP or IL10 were injected into the tail vein. Mice were killed after an additional 24 h, the bronchoalveolar lavage fluid (BALF) was collected, lungs were digested and the immune composition analyzed by flow cytometry analyzed. **A** Mouse weight loss during experiment. **B** Number of cells in the BALF. **C** Tnf-α concentration in the first 1.5 mL of the BALF quantified with ELISA. **D** Frequency of Cd19 + cells in Cd45 + cells. **E** Frequency of Cd3 + Cd8 + cells in Cd45 + cells. **F** Frequency of Cd3 + Cd4 + cells in Cd45 + cells. **G** Frequency of Cd11b + Cd11c + cells in Cd45 + cells. **H** Frequency of Cd11b + Ly6g + cells in Cd45 + cells. Depicted are the means. Errors bars indicate SD. Statistical significance was calculated with Two-way-ANOVA and subsequent Bonferroni-corrected pairwise *t*-test. *: *p* < 0.05, **: *p* < 0.01, ***: *p* < 0.001

Next, we tested whether the CXCL11S1-IL10-transduced MSCs (IL10-MSCs) show superior immune-modulation compared to CXCL11S1-eGFP-transduced MSC (eGFP-MSC) when tested in an LPS-induced ALI mouse model [45]. MSCs are entrapped in the lung upon intravenous injection in the first hours [46]. We assume that the therapeutic effect of MSC application should not depend on their migration potential into the lung. However, we do not know how many MSCs actively pass the alveolar–capillary barrier and whether this is necessary for their therapeutic effect. Intratracheally applied LPS (5 µg or 10 µg in 50 µL) led to weight loss, an increase of Tnf-α and inflammatory cells in the BALF within 28 h (Additional file 8). The intravenous injection of PBS, eGFP-MSC, or IL10-MSCs 4 h post-LPS application showed no significant effect on body weight, the number of immune cells, or Tnf-α concentration in the BALF within 24 h (Fig. 4A–C). Next, we assessed the different immune cell types. The transplantation of CXCL11S1-IL10 MSCs increased the percentage of Cd45 + Cd19 + B-cells compared to PBS or CXCL11S1-eGFP-MSC experimental groups (Fig. 4D). The injection of CXCL11S1-IL10 MSCs did not affect the abundance of Cd3 + Cd8 + cytotoxic T-cells but increased the percentage of Cd3 + Cd4 + T-helper cells compared to the PBS group (Fig. 4E-F). We observed no effect on the percentage of Cd11b + Cd11c + macrophages (Fig. 4H) but measured a reduced number of Cd11b + Ly6g + neutrophils in the 10 µg/IL10-MSC group compared to PBS or eGFP-MSCs. (Fig. 4I).

## Discussion

MSCs are used in hundreds of clinical trials for decades, but only a few therapeutic cell products were approved by regulatory authorities, as the beneficial effects were most often moderate [2–4]. Many work groups test genetic modifications of MSCs to improve their therapeutic potential. However, constant transgene expression, like immune-inhibitory factors, in ectopic organs can lead to an increased risk of infections and might be unnecessary in the target organ, if the disease occurs in waves [12, 13]. In this work, we show several inflammation-inducible promoters which were tested in vitro and in an ALI mouse model. Furthermore, we suggest CD317 as a reliable marker for immune activation of MSCs.

MSCs exert most of their immune-modulatory effects upon contact with a pro-inflammatory microenvironment [14]. A clinical study correlated the response to MSC treatment to a transient increase in IFN-γ levels [47]. Since an inflammatory activation seems to be necessary to induce immune modulation, we decided to exploit physiologically activated promoters to drive potential transgenes of interest, here IL10. However, as these regulatory elements can be very complex and distributed among thousands of base pairs [17], we amended our candidate list of promoters from inflammation-induced genes by synthetic promoters. The combination of GC-box, CAAT-box, BRE, TATA-box, MTE and DPE in the ECA7 minimal promoter showed no background expression and a reliable and high activation upon the combination with IFN-γ-responsive elements (GAS, ISRE). The GAS element was more dependent on the presence of all elements than ISRE, which reached comparable transgene levels without MTE/DPE or GC/CAAT-box. In our experiments, the ECA7 minimal promoter showed a lower donor variability than a previously published synthetic TATA-box [34]. As we controlled for the transduction with constitutively transcribed mCherry and we tested different vector amounts, transduction efficiency as an explanation is very unlikely. Another explanation for the observation could be different cell types or cell states. Haberle and colleagues discussed that the dependency on certain core promoter elements is different among transcribed genes and, consequently, their regulatory elements [15]. We showed in previous studies that MSCs are subjected to a massive clonal selection, and selected clones show different abilities [21, 48]. Kwee and colleagues showed heterogeneity among MSCs upon IFN-γ treatment [49]. The ECA7 promoter might reduce the transgene expression variability among upstream transcription factor binding sites (GAS/ISRE) or donors by providing several general transcription factor binding sites.

The identification of physiological promoters can be very challenging, as our data confirmed. Both IDO promoter variants showed nearly no transgene expression upon pro-inflammatory activation, and the CD317 promoter showed a high background activity without activation. We showed with our antibody staining that CD317 was only detected on the surface upon activation of MSCs. Our previous study showed the regulation on mRNA level [19]. We had problems during the cloning of the promoter, as a GC region prevented amplification from the genome or assembly by a commercial provider, so we had to test a shorter version. By shortening the promoter, we might have removed negative regulatory elements, such as binding sites of CTCF with a GC-rich binding motif [50]. Since CD317 expression correlated with one of the most prominent MSC effector proteins IDO1, we used CD317 as a simple activation marker. This finding is additionally interesting, as in future studies, CD317 can be used for the sorting of living cells, in contrast to fixation of cells for intracellular IDO1 detection. Among the inducible promoters (GECA7, IEAC7, CXCL9, CXCL10, and CXCL11), the CXCL11 promoter showed the highest and broadest pro-inflammatory activation profile. The activity increased upon IFN-γ and TNF-α, as well as an IL1-β treatment in combination with IFN-γ. We designed shorter versions to characterize the CXCL11 promoter and narrowed the regulatory activity to 300 bp at the 3´end. Only in this region, we found inflammation-regulated NFκB and AP-1 family binding sites [37, 38]. The 300 bp CXCL11S1 version was sufficient for in vitro IL10 secretion and to achieve significant differences in an ALI mouse model. We chose IL10 as proof-of-concept, as it is used in many disease contexts [6, 39, 40, 42, 43, 51] and it showed no effect on MSCs in our transcriptome analyses. However, the benefit of additional IL10 expression by transplanted MSCs in the Ali mouse model was shown only for some of the assessed parameters. We observed no effect on weight loss, cell numbers in the BALF, or abundance of Cd3 + Cd8 + or Cd11b + Cd11c + . We observed only a trend of decreased Tnf-α concentration in the BALF and a significantly higher percentage of Cd19 + B-cells and Cd3 + Cd4 + T cells, and a significantly reduced number of Cd11b + Ly6g + myeloid cells. Whether our observations would translate to a therapeutically relevant effect in patients is difficult to judge. Zhu and colleagues transplanted unmodified UC-MSCs into patients suffering from COVID-19 infection in a placebo-controlled clinical trial [52]. MSC infusion shortened the time to symptom remission compared to the placebo group. Interestingly, the authors observed an increase of monocytes and a decrease of B cells and CD4 + T cells in the peripheral blood of MSC-transplanted patients. They analyzed the immune cell composition in the peripheral blood and the lungs of an ALI mouse model. They showed an increase of B cells in the lung of MSC transplanted mice compared to the LPS-only group. The investigators hypothesized that the reduced proportion of B cells in the peripheral blood was due to the influx of B cells into the lung. Similarly, we observed an increase in B cells in mouse lungs only in the CXCL11S1-IL10-MSC and not in the PBS or CXCL11S1-eGFP-MSC group. Subsequent optimization of the MSC application route, MSC therapeutic time window, analysis time point, assessed parameters and an evaluation of other transgenes will contribute to a better understanding of observed effects in the mouse model.

## Conclusion

We could show that MSCs express CD317 upon inflammatory activation, which marks IDO1-expressing MSCs. This surface marker could be used as an MSC activation marker. Further, we designed and evaluated the minimal promoter ECA7 with low background activity and reliable activation by IFN-γ addition. The described minimal promoters could be combined with other pathway-specific DNA motif elements to exploit other signaling pathways in other research fields. Conditional expression provides the possibility to limit the transgene effect to a certain microenvironment. For many inflammation-driven diseases and cell types, which potentially engraft for years in patients, such as regulatory T cells, transgene expression is only needed upon reoccurring inflammation [53]. We also showed that a shortened version of the CXCL11 promoter is broadly activated in an inflammatory context, sufficient to drive IL10 secretion. In our handy, IL10 was not naturally secreted by UC-MSCs and had no effect on the transcriptome of MSCs. However, it potentially improved MSC effects in an ALI mouse model. In summary, our results provide many insights into MSC activation and conditional transgene expression, which can be transferred to other disease contexts and cell types.

### Supplementary Information

**Additional file 1: **The influence of CXCL11S1-driven IL10 overexpression by UC-MSCs in an ALI mouse model.

**Additional file 2:** Gating strategy of MSCs transduced with lentiviral vectors.

**Additional file 3: **Confirmation of the mesenchymal origin by surface staining.

**Additional file 4:** Statistical analysis of promoter activation

**Additional file 5:** Analyses of GAS or ISRE elements combined with different minimal promoters.

**Additional file 6: **Antibody staining of CD317 and IDO1 upon different stimuli.

**Additional file 7: **Detailed overview of transcription factor binding position.

**Additional file 8:** The effect of the ALI mouse model without a therapeutic attempt.

## Data Availability

The microarray data can be accessed in the Gene Expression Omnibus database (GSE224735). Detailed plasmid sequences are available upon request.

## Abbreviations

ALI: Acute lung injury
ARDS: Acute respiratory distress syndrome
BALF: Bronchoalveolar lavage fluid
BRE: TFIIB recognition element
CD: Cluster of differentiation
CXCL10: CXC-Ligand 10
CXCL11: CXC-Ligand 11
CXCL9: CXC-Ligand 9
CXCR4: C-X-C chemokine receptor type 4
DPE: Downstream promoter element
ECA: Enhanced core promoter A
EFS: Elongation factor 1 α short promoter
eGFP: Enhanced green fluorescent protein
GAS: Gamma interferon activation site
IFN-γ: Interferon-γ
IL10: Interleukin 10
IL1-β: Interleukin 1 β
ISRE: Interferon-sensitive response element
MSC: Mesenchymal stromal cell
MTE: Motif ten element
PGK: Phosphoglycerate kinase
SFFV: Spleen focus forming virus
TNF-α: Tumor necrosis factor α
UC: Umbilical cord
UCP: Umbilical cord piece
