# Supplementary material for: Inflammation-inducible promoters to overexpress immune inhibitory factors by MSCs
Source: Stem Cell Res Ther. 2023 Sep 23;14:270. doi: 10.1186/s13287-023-03501-6 (PMC10518110; doi:10.1186/s13287-023-03501-6)
Supplement: Supplementary file 2 — Additional file 2: Gating strategy of MSCs transduced with lentiviral vectors. [file 13287_2023_3501_MOESM2_ESM.docx]

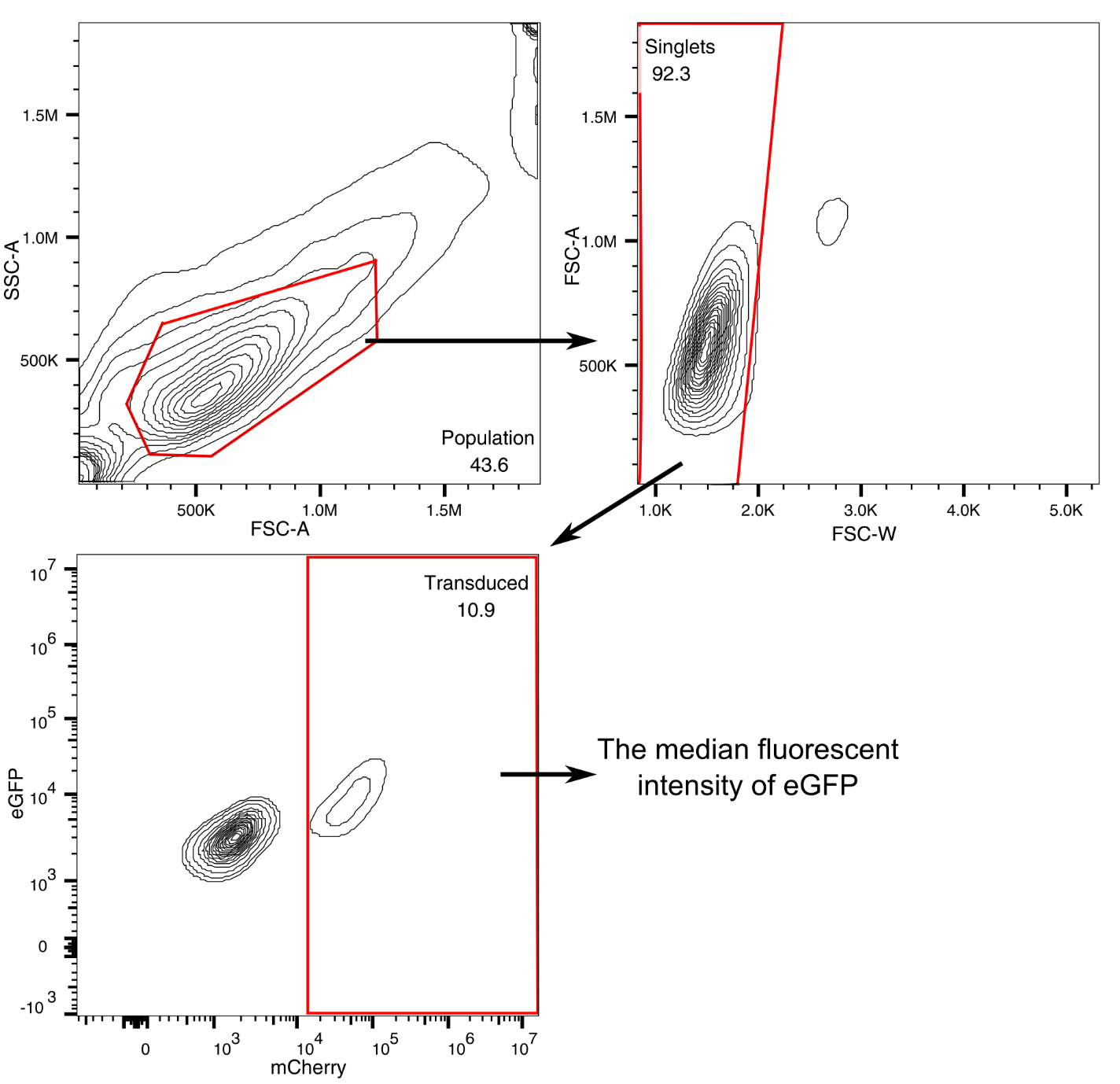


**Supplementary File 2. Gating strategy of MSCs transduced with lentiviral vectors.**

The forward and side scatters were used to discriminate MSCs (population gate) from debris. The forward scatter area in dependency of forward scatter width was used to distinguish single cells (Singlets) from doublets. Finally, the median fluorescent intensity of eGFP of mCherry-positive MSCs was determined.
