# Supplementary material for: Inflammation-inducible promoters to overexpress immune inhibitory factors by MSCs
Source: Stem Cell Res Ther. 2023 Sep 23;14:270. doi: 10.1186/s13287-023-03501-6 (PMC10518110; doi:10.1186/s13287-023-03501-6)
Supplement: Supplementary file 3 — Additional file 3: Confirmation of the mesenchymal origin by surface staining. [file 13287_2023_3501_MOESM3_ESM.docx]

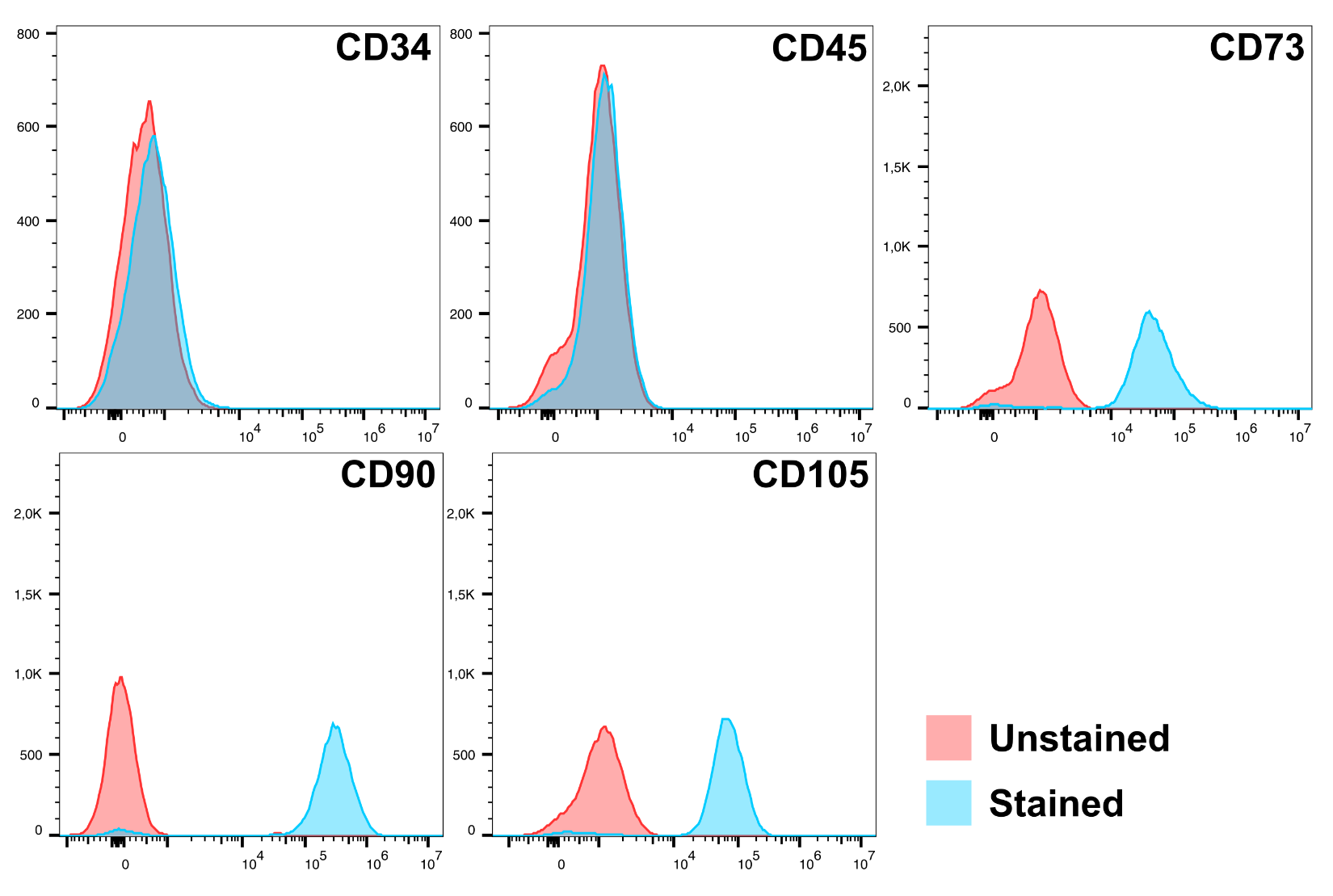


**Supplementary Figure 3. Confirmation of the mesenchymal origin by surface staining**

Human umbilical cord pieces were cultivated in MSC15 (MEM α, GlutaMAX™ Supplement, no nucleosides, 15% hAB-serum, 1% penicillin/streptomycin) for approximately 2 weeks until cells grew out. Cells were passaged at least one time to enable the outgrowth of MSCs of other cells.
