# Supplementary figures and images for: Inflammation-inducible promoters to overexpress immune inhibitory factors by MSCs
Source: Stem Cell Res Ther. 2023 Sep 23;14:270. doi: 10.1186/s13287-023-03501-6 (PMC10518110; doi:10.1186/s13287-023-03501-6)

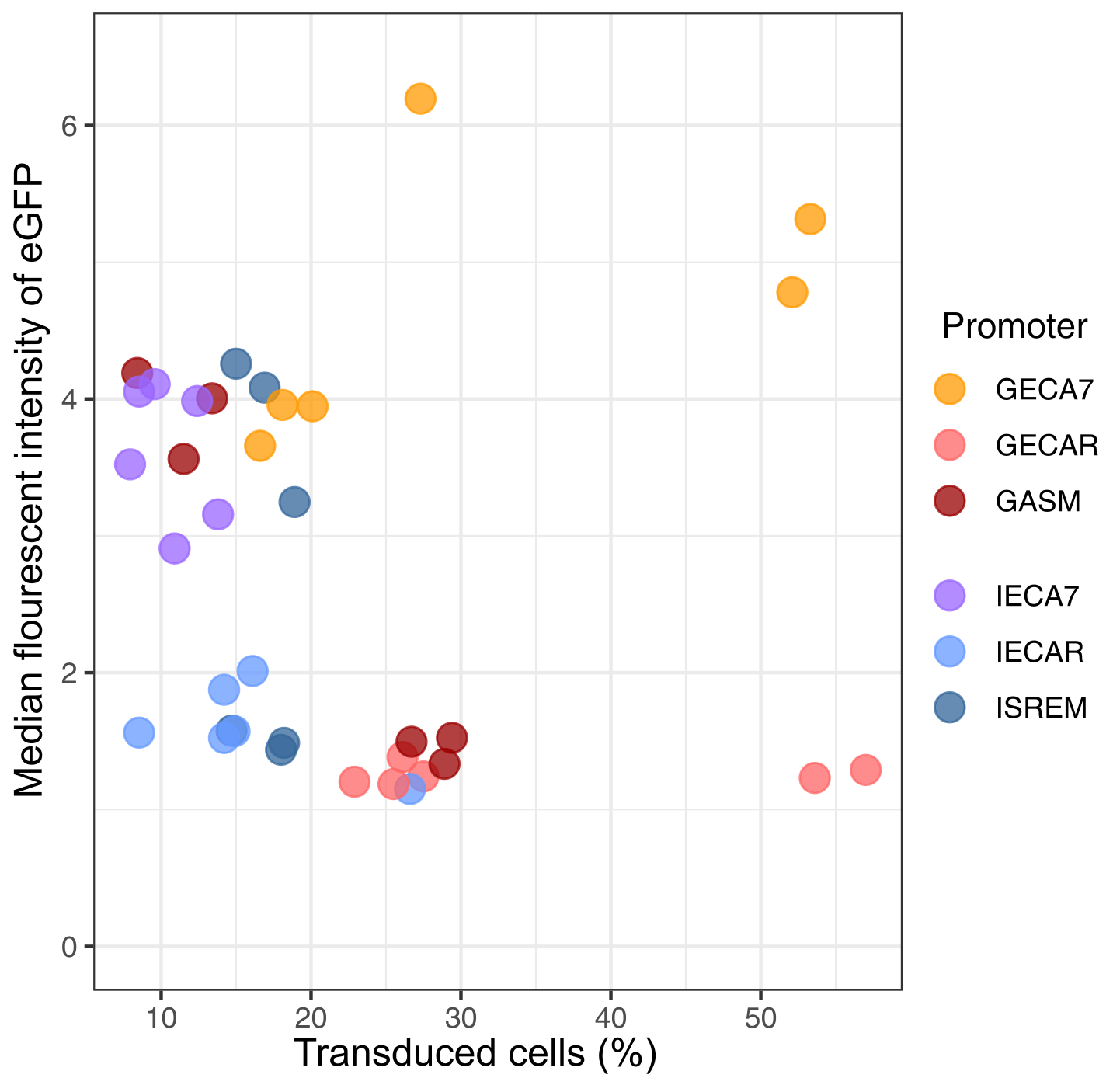

Supplement: Supplementary file 4 — Additional file 4: Statistical analysis of promoter activation [file 13287_2023_3501_MOESM4_ESM.png]
