# Supplementary material for: Inflammation-inducible promoters to overexpress immune inhibitory factors by MSCs
Source: Stem Cell Res Ther. 2023 Sep 23;14:270. doi: 10.1186/s13287-023-03501-6 (PMC10518110; doi:10.1186/s13287-023-03501-6)
Supplement: Supplementary file 6 — Additional file 6: Antibody staining of CD317 and IDO1 upon different stimuli. [file 13287_2023_3501_MOESM6_ESM.docx]

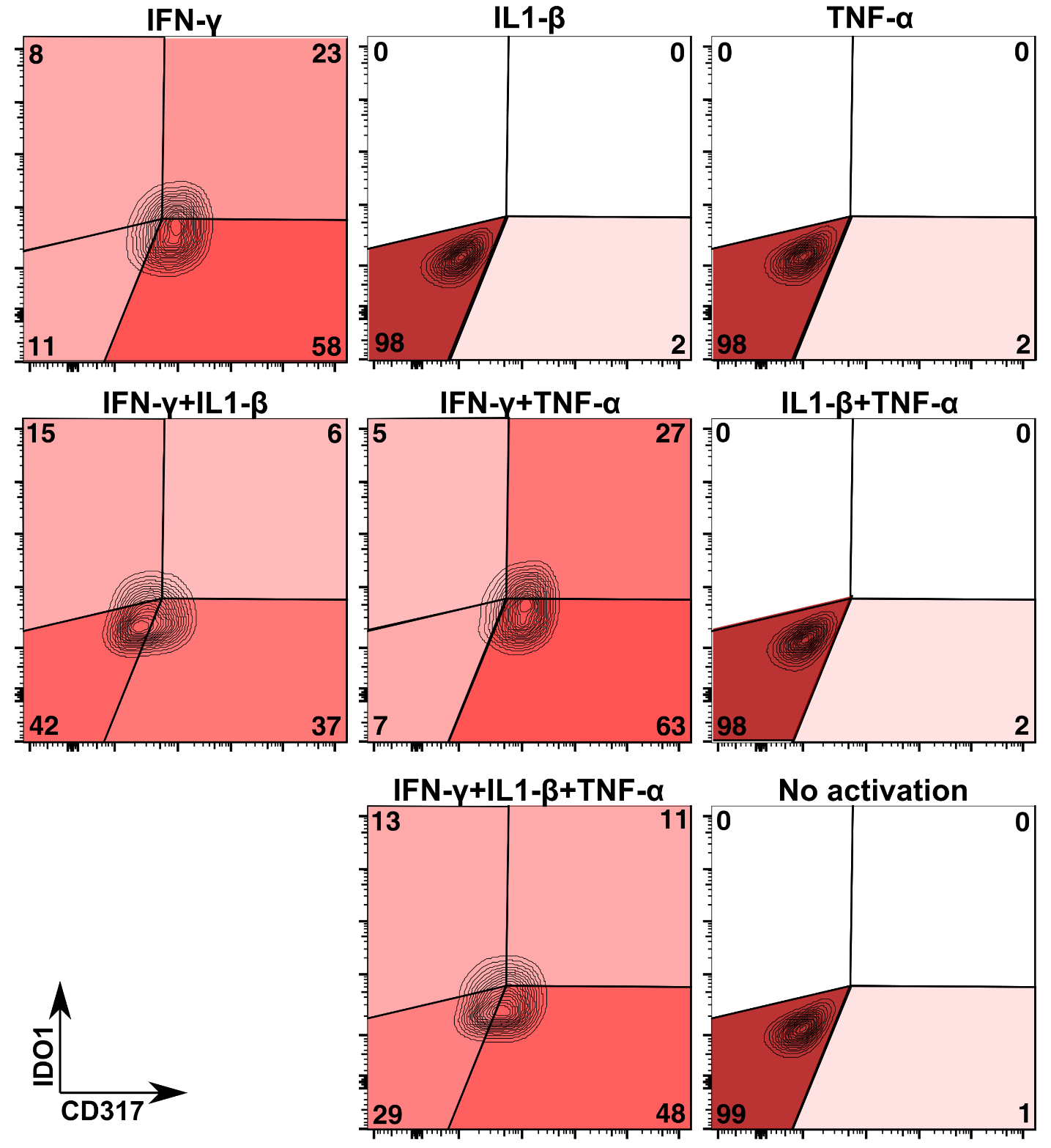


**Supplementary File 6. Antibody staining of CD317 and IDO1 upon different stimuli**

Human UC-MSCs were treated with different combinations of 25 ng/mL IFN-γ, IL1-β and TNF-α. After 24 h, the surface CD317 and intracellular IDO1 proteins were stained with antibodies and gating was set with unstained MSCs. For a better overview, backgrounds were colored in different shades of red dependent on percentage of cells in the gates.
