# Supplementary material for: Inflammation-inducible promoters to overexpress immune inhibitory factors by MSCs
Source: Stem Cell Res Ther. 2023 Sep 23;14:270. doi: 10.1186/s13287-023-03501-6 (PMC10518110; doi:10.1186/s13287-023-03501-6)
Supplement: Supplementary file 8 — Additional file 8: The effect of the ALI mouse model without a therapeutic attempt. [file 13287_2023_3501_MOESM8_ESM.docx]

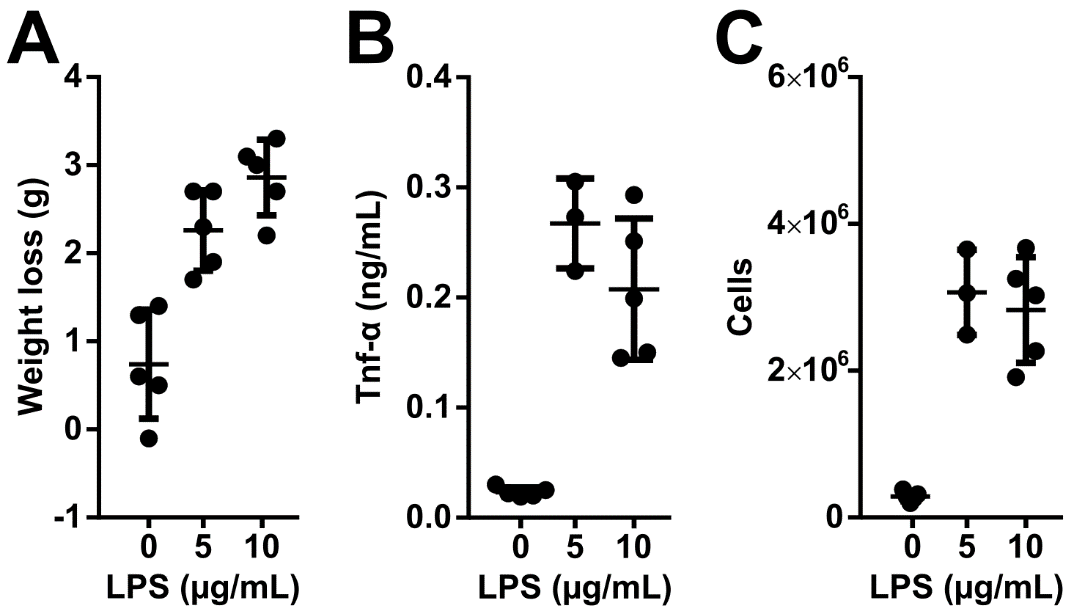


**Supplementary File 8. The effect of the ALI mouse model without a therapeutic attempt**

C57BL/6J mice were intratracheally installed with 50 µL of 0, 5 µg/mL or 10 µg/mL LPS in PBS to induce ALI. After 4 h, PBS was injected into the tail vein, mice were sacrificed after additional 24 h and the bronchoalveolar lavage fluid (BALF) collected. **(A)** Mouse weight loss during experiment. **(B)** Tnf-α concentration in the first 1.5 mL of the BALF was quantified with ELISA. **(C)** Number of cells in the BALF.
